# Modelling the significance of health values, beliefs and norms on the intention to consume and the consumption of organic foods

**DOI:** 10.1016/j.heliyon.2023.e17487

**Published:** 2023-06-20

**Authors:** Qing Yang, Abdullah Al Mamun, Farzana Naznen, Long Siyu, Zafir Khan Mohamed Makhbul

**Affiliations:** a UKM - Graduate School of Business, Universiti Kebangsaan Malaysia, 43600, UKM Bangi, Selangor Darul Ehsan, Malaysia; b UCSI Graduate Business School, UCSI University, Cheras, 56000, Kuala Lumpur, Malaysia

**Keywords:** Consumption of organic food, VBN theory, Health value and consciousness, Trust in organic food

## Abstract

The present research aims to extend the value-belief-norm model by including health values, health consciousness, healthy eating beliefs, and trust in organic food as the impelling factors. This study empirically tested the holistic framework to understand the important factors in consumers’ decision-making processes concerning organic food consumption. A web-based survey was performed to collect data from a convenience sample of 571 organic food consuming university students in China. The hypotheses were tested using partial least square structural equation modelling (PLS-SEM). Based on the findings, health values and health consciousness had substantial impacts on healthy eating beliefs, which in turn positively affected personal norms and awareness of consequences. Additionally, awareness of consequences and ascription of responsibility had major effects on personal norms. Likewise, personal norms and trust in organic food had a profound influence on the intention to consume organic foods, which in turn significantly induced actual consumption. The findings not only provide novel insights for researchers to understand the aspects of organic food consumption but present a guideline for marketers to develop appropriate marketing tactics to grow the organic food business. This study recommends that policymakers should focus on increasing the awareness and knowledge of organic food, encouraging organic food production, and prioritising campaigns showcasing the unique health benefits of organic food to stimulate increased consumption.

## 1 Introduction

Food consumption habits are constantly evolving due to environmental concerns, nutritional concerns, and health hazards [1]. According to the World Health Organization [2], in 2020, 41 million deaths caused by non-communicable illnesses, with unhealthy diets being one of the primary causes, were recorded worldwide. Environmental security and food safety incidents throughout the world have raised consumer health awareness in recent times, causing organic food to become a popular topic of discussion [3]. The new lifestyle catastrophe (physical inactivity) that results in a higher risk for heart disease and metabolic syndrome, which may cause diabetes and a raise in numerous types of cancer, is driving the increase in the demand for organic foods while decreasing the consumption of conventional foods [4]. Therefore, worldwide food safety and food-related environmental issues have gained more attention [5].

The significant interest of consumers in organic food over the past two decades is evident by the gradual increase in global demand for organic food [3,6,7]. The global sales of organic food are estimated to have surpassed USD 90 billion in the last 20 years [8]. Furthermore, a report shows that as of 2019, 80% of global production was concentrated in developing countries, with Asia having the highest number of organic food crops and ranking third in global sales [7]. As of 2021, the sales of organic products in China reached USD 4.8 billion, accounting for about 8% of global demand, but the per capita consumption was only USD 3.4 [9]. Based on these data, there is still room to promote organic food consumption in China, as China's per capita consumption level still significantly lags behind many other developed economies [5].

Numerous life assessments have proven the ecological benefits of consuming organic foods, namely evaluating factors such as eco-diversity, non-toxicity, and soil fertility [10]. Organic foods are frequently deemed eco-friendlier and, as a result, healthier than traditional, non-organic diets [1]. Furthermore, organic food items are attributable to improved food quality and health due to lower agrochemicals, pesticides, and heavy metal levels compared to traditional foods [10,11]. Organic food is considered an essential type of sustainable consumption [12], since it is healthier and more eco-friendly than conventional food and may benefit the local economy [3]. Although organic foods supersede conventional foods in the context of health [13], the current verification techniques of organic foods’ do not persuade all the customers to buy them [14]. Considering organic foods not only provide considerable health benefits but also have an apparent pro-environmental feature, marketers, producers, and academics must have a broader understanding of consumer behaviours [15]. This necessitates the identification of factors that might encourage consumers to become more actively involved in organic food intake.

Examining the influence of organic food features on purchase intention has gained considerable scholarly attention [14,16]. Literature on organic food is widely available in a diverse range of scopes, countries, and audiences, and it is recommended that more rigorous research is conducted based on various contexts and circumstances [3,6,7,17,18]. This present study has identified a population gap in the literature that focuses on China's young generation and their perceptions of organic foods. Thus, to address this demographic gap and consider the recommendations of previous researchers, this study has selected university students aged 18–35 years old in China as the target population to identify the major factors that influence their consumption of organic foods. The current study also highlights three major theoretical gaps in the literature on organic food consumption behaviour. First, the internal/intrinsic factors, including health values and healthy eating beliefs, that drive consumers' decision making regarding organic food are inadequately conceptualised. Second, the norm relevant aspects, such as personal norms and trust in organic food, that are associated with consumers' intention to consume organic foods remain unclear. Finally, the behavioural outcomes with the empirical analysis based on the value-belief-norm (VBN) theory have rarely been examined in existing literature regarding the consumption of organic foods. Hence, this study aims to address these gaps by enhancing the current understanding of organic food consumption practices among consumers by proposing and testing an extended VBN theory. More precisely, this study proposes a holistic research framework for analysing the intention to consume organic foods by including health values, healthy eating beliefs, and trust in organic food as additional impelling factors in the original VBN model. The study empirically evaluates the association between values, beliefs, and personal norms to gain a complete understanding of consumers' decision-making processes regarding organic food. The current study attempts to answer three major research questions, namely: (1) what is the relationship between consumers' health values and health consciousness and their healthy eating beliefs? (2) how are healthy eating beliefs, awareness of consequences, and ascription of responsibility associated with personal norms? and (3) how do personal norms and trust in organic food influence the intention to consume organic foods, and in turn actual consumption?

The rest of this paper is divided into six sections. Section 1 provides an overview of the literature as well as the theoretical foundations and the study's hypotheses. Following that, Section 2 describes the methodological approaches used, while Section 3 discusses the data analysis and findings. Next, Section 4 contains a detailed discussion of the research findings whereas Section 5 discusses the theoretical and practical implications based on the research findings. Lastly, in Section 6, a brief overview, study limitations, and recommendations for future research are presented.

## 2 Literature review

### 2.1 Theoretical foundation

**Fig. 1:**
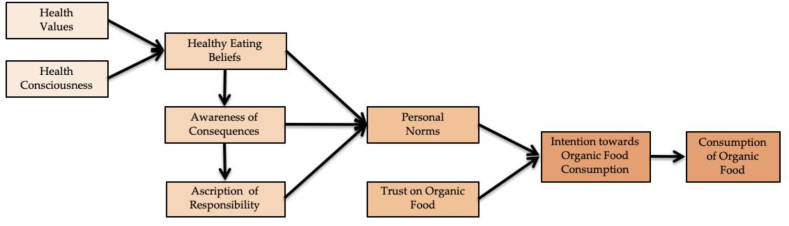
Research framework.

Numerous theories have been used in the investigation of organic food consumption, and among those that are most researched, well documented, and well-represented are the Theory of Planned Behaviour (TPB) [12,19,20] and the Stimulus-Organism-Response (S–*O*-R) [5,21]. On the other hand, the VBN theory is based on values that explain environmentalism and are expressed to various degrees in individuals [22]. Stern et al. [23], have coined the VBN theory which interconnects five factors explaining an individual's behaviour (i.e., personal values, awareness of consequences, ascription of responsibility, personal norms, and behavioural intention) in a chain of events to anticipate pro-environmental behaviour. It shifts from the consistent factors of an individual's personality and beliefs to focus on the adverse consequences and building responsibility for reducing potential risks [24]. The VBN theory is commonly used in the context of pro-environmental and eco-friendly decisions, including the purchasing of a variety of green items [20,[25], [26], [27]]. Consumers may purchase and consume organic foods for a variety of reasons, such as health benefits, good taste, animal protection, and effects on the environment [1,14], personal beliefs, functional values, and trust [7], and social identities and motivating factors [28]. Given that organic food consumption is regarded as an ecologically sustainable and green purchasing behaviour [20], it is more logical to associate it with socio-psychological aspects relating to individual values and norms. Thus, the current study adopts the VBN theory as the appropriate theoretical base and expands it with health values, health consciousness, healthy eating beliefs, and trust (Fig. 1) to explain the behavioural and decision-making characteristics in the context of organic food consumption.

### 2.2 Hypotheses development

#### 2.2.1 Health values (HV)

Values vary in priority and can be considered individuals' desired aims that become the guiding ideologies in their lives [29]. People's proactive behaviour to better their health, both now and in the future, is defined as health as a personal value [17]. Health as a value has been explicitly assessed by individual views in which people believe that good health is the most essential aspect of a happy life and they place a high priority on achieving a healthier life [30]. The study of health values in the context of organic food employs two key aspects, namely personal health apprehension that promotes healthy consumption beliefs and health features of organic food [17]. Past research reports that the healthy ingredients of organic foods influence customers' personal values which in turn favourably impacts their purchasing behaviours [4,31]. Hence, customers who value a healthy life may believe that organic products can improve their overall healthy eating habits since these products utilise natural ingredients [10]. Health values have been established as a major motivator in determining organic purchase behaviour, specifically organic food items [4,32]. Thus, the following hypothesis is proposed.

H1 Health Values has a positive effect on Healthy Eating Beliefs.

#### 2.2.2 Health consciousness (HC)

Health consciousness indicates people's views towards health problems and their willingness to take measures to preserve their health [33]. Furthermore, it relates to individuals' perception of improvements in their health condition and the level of attention placed on health care [34]. Consumers may avoid consuming products containing chemical substances, and instead, increase the use of products offering health advantages [35]. According to customers, the most significant feature of organic foods is healthiness [36]. In general, organic foods are preferred by health-conscious individuals because, in addition to being safe and nutritious, organic foods are regarded to be chemical-free, devoid of additives, and eco-friendly [6], resulting in fewer health issues. Therefore, health consciousness is a major indicator of organic food consumption [17,37] which develops healthy eating beliefs. Recent studies have shown that consumers are increasingly becoming health conscious, which is boosting their beliefs and preferences for natural and healthy food consumption [15,19]. A study by Iqbal et al. [12] has identified that consumer health consciousness is favourably associated with their active participation in healthy food consumption, which promotes higher purchase intentions. These arguments lead the current study to hypothesise as follows.

H2 Health Consciousness has a positive influence on Healthy Eating Beliefs.

#### 2.2.3 Healthy eating beliefs (HE)

The most prevalent manifestation of healthy eating is the consumption of fresh and organic fruits and vegetables [38,39]. Organic food intake has high moral significance due to the general concern for the environment that is linked to organic food [10]. Individuals’ awareness of the negative repercussions of non-organic food intake improves as a result of these beliefs. For instance, numerous deadly diseases and childhood illnesses are rising, and many people believe these illnesses are connected to environmental issues, pesticides, and food chemicals [7]. Therefore, individuals feel morally obliged to embrace organic food to improve their healthy eating habits while at the same time protecting the environment from the harmful effects of non-organic food manufacturing chemicals. Hence, the current study postulates the following hypotheses concerning healthy eating beliefs, awareness of consequences, and personal norms.

H3a Healthy eating beliefs has a positive influence on awareness of consequences.

H3b Healthy eating beliefs has a positive influence on personal norms

### 2.3 Awareness of consequences (AC)

Individuals' perspectives of the favourable consequences of any activity on others are defined as awareness of consequences [40]. A positive association exists between AC and ascription of responsibility [41] and AC activates personal norms [42,43]. Al Mamun et al. [44] found that in terms of energy conservation, an enhanced awareness of consequences positively influenced a sense of responsibility for reducing individual climate impact through the adoption of environmentally friendly transportation modes, with this responsibility attributed to the formation of personal energy-saving norms. Meanwhile, Duong et al. [45] report that farmers who intend to embrace organic agriculture are aware of the advantages of organic farming and believe they are personally liable for the environmental implications of using agrochemicals. Farmers are also shown to have an ethical obligation to employ organic practices to safeguard and promote consumers’ health [41,46]. Besides, there is a belief that if a person becomes aware of the adverse consequences of consuming non-organic foods, he or she will feel morally obligated to adopt organic foods [17]. As such, the following hypotheses are developed.

H4a Awareness of consequences has a positive effect on ascription of responsibility.

H4b Awareness of consequences has a positive effect on personal norms.

#### 2.3.1 Ascription of responsibility (AR)

A sense of personal accountability due to pro-social activities is reflected through the ascription of responsibility [40]. AR is an individual's belief that his or her efforts may prohibit any adverse consequences from occurring [47]. Individuals who believe they are accountable for their activities and/or being inactive and are aware of the consequences of their actions and/or inaction are more prone to participate in pro-environmental activities that are consistent with their personal values [22]. Han [48] has revealed that AR has a substantial impact on personal norms to participate in eco-friendly activities. It is normal for people to acquire moral obligations when they acknowledge their responsibilities for the adverse repercussions of their actions. Nevertheless, if individuals deny their responsibility for the resulting negative effects, personal norms are unlikely to arise [44]. Based on their research on organic milk purchase, Roos and Hahn [42] report a significant association between personal norms and AR. In the context of environmental citizenship behaviour, personal norms are found to be positively affected by ascription of responsibility [49]. Considering the substantial impact of AR on personal norms in pro-environmental contexts, this study proposes a similar association in the context of organic food consumption. Hence, the following hypothesis is formed.

H5 Ascription of responsibility has a positive influence on personal norms.

#### 2.3.2 Personal norms (PN)

Personal norms decide whether a person should engage in an activity to prevent undesirable consequences [47]. Moreover, personal norms trigger when a person believes that a social initiative positively influences another individual and when that person feels accountable for the adverse consequences due to his or her inactivity [41]. In the context of organic farming, Fatemi and Rezaei-Moghaddam [24] explain moral norms as societal standards as well as producers' self-expectations of demonstrating eco-friendly behaviours as a moral obligation, such as avoiding the use of agrochemicals. On the other hand, consumers respond in ways that are consistent with their personal norms while purchasing organic milk [42]. Personal norms are an important element that directly influences behavioural intention and pro-environmental activity [50,51]. In another organic farming study, Nguyen et al. [41] have identified that personal norms interacts as a strong mediator in the relationships between farmers' intention to practice organic food and awareness of consequences and ascription of responsibility. According to Prakash and Pathak's [52] study, environmentally friendly packaging is the priority among Indian consumers due to strong moral norms to minimise environmental adversity. As such, this study proposes the following hypothesis since strong personal norms can drive consumers' intention to consume organic foods.

H6 Personal norms have a positive influence on the intention to consume organic foods.

#### 2.3.3 Trust in organic food (TOF)

Trust is described as consumers' perception of trustworthiness evaluation while purchasing organic foods [53]. Many customers have concerns about the promises made by organic food manufacturers [54], hence, trust is critical in influencing consumers' decision to purchase organic foods [55]. According to Shahabi and Gorton [56], food safety, government laws and regulation, and proper labelling impact the intention to purchase organic foods. Zhang et al. [57] assert that when customers believe in organic labelling, they are more likely to buy organic foods. Organic labelling seems to be a technique employed by the government to ensure the quality of organic foods [58]. Consumers regard certification and labelling as secondary indicators that extend their trust in organic foods [53]. A crucial trust criterion for organic foods is their health content [10]. Organic foods' health content encompasses safety, quality, nutritious elements, flavour, etc., resulting in customers' favourable opinion towards such items [7]. Given the extrinsic advantages of the organic food production methods, ethical influence on organic food may improve consumers’ environmental consciousness, which is one of the motivating elements for customers to trust organic foods [59]. Organic food manufacturing procedures achieve balance through eco-friendly cultivation and packaging, which strengthens customer trust and, in turn, purchasing behaviour [1,60]. Therefore, this study has formulated the following hypothesis.

H7 Trust in organic foods has a positive effect on the intention to consume organic foods.

#### 2.3.4 Intention to consume organic foods (IOF) and consumption of organic foods (COF)

Behaviour is defined as an intent-driven activity performed due to internal processes [61]. The intention to perform an activity is a determining factor in the explosion or non-explosion of a certain behaviour [62]. Human behaviour is shaped by intention because a person is regarded to be rational and as someone who intends to achieve a certain objective and then behaves accordingly [63]. In the context of organic farming, human behaviour concerns a farmer's goal to prepare for the elimination of all hazardous and chemical additives in agricultural production and the adoption of organic agriculture [24]. On this basis, the intention to practice organic agriculture has a positive and strong association with the adoption of organic agriculture. Hence, the current study proposes the following hypothesis.

H8 Intention to consume organic foods has a positive effect on the consumption of organic foods.

All the hypothesised associations are presented in Fig. 1.

## 3 Research methodology

### 3.1 Population and data collection

A cross-sectional non-experimental design was used, with a quantitative method i.e. a survey, to evaluate the proposed research model. This study selected university students in China aged 18–30 years old as the intended audience. They belong to the customer group that typically makes purchase decisions and are deemed to be familiar with the advantages of organic food. This study created an online survey questionnaire using the Google form, and the survey link was distributed across different universities with the assistance of volunteer students. The recommended sample size was calculated to be 160 using the G-Power software along with an effect size of 0.15, statistical power of 0.95, and α-value of 0.05, for a total of eight predictors [64]. Nonetheless, data were obtained from 571 students to eliminate any potential conflicts induced by a small number of respondents. Participation in this study was fully voluntary, and participants were assured that their personal information would be treated confidentially. The human research ethics committee of Changzhi University approved this study (approval number: CZ-2022-0076). Written informed consent was obtained from respondents who participated in the survey.

### 3.2 Survey instrument

The questionnaire was developed based on instruments validated in past studies, with a few minor adjustments relevant to the present study. The four items that tested Health Values were adopted from Lau et al. [30] whereas health consciousness was evaluated by adopting measures from Yang et al. [33]. Next, healthy eating beliefs was assessed using five items taken from Han, Hwang and Lee [48]. Awareness of consequences and ascription of responsibility were both gauged using items from Stern et al. [23]. To evaluate personal norms, five items were taken from Choi et al. [65] and Ünal et al. [25]. Meanwhile, trust in organic food was measured using five items derived from the study of Chen [66] and items to assess the intention to consume organic foods were adopted from Chen and Deng [67] and Maichum et al. [68]. Finally, consumption of organic foods was evaluated using items from Walton and Austin [69] and Sánchez et al. [70]. For all the constructs, the questionnaire was developed using a five-point Likert scale. For respondents to feel engrossed while answering, the questionnaire was crafted using neutral and comprehensible wordings. All items used in this study presented in Supplementary Material S1. Survey Instrument.

### 3.3 Common method bias

To investigate the existence of common method variance issue, this research employed Harman's single-factor test, a frequently used method, to ensure that the study model was not significantly influenced by common method bias [71]. The single component explained 42.76%

of the variation, which was less than the 50.00% threshold recommended by Podsakoff et al. [72], confirming that common method bias was not an issue for the current study's data. Additionally, as recommended by Sarstedt et al. [73], a full collinearity test was conducted for all the components and the variance inflation factor (VIF) values were: 1.975 (health values), 2.489 (health consciousness), 2.501 (healthy eating beliefs), 3.619 (awareness of consequences), 2.824 (ascription of responsibility), 2.964 (personal norms), 3.220 (trust in organic food), 3.493 (intention to consume organic foods), and 3.735 (consumption of organic foods). Since all the components had VIF values less than the maximum threshold of 5, as specified by Kock and Lynn [74] and Hair et al. [75], it was determined that the single-source data were not skewed and there was no evidence of common method bias.

### 3.4 Multivariate normality

This study utilised a statistical tool from the Web Power website to examine the multivariate skewness and kurtosis to assess multivariate normality. The results indicated that the data were not normally distributed because multivariate skewness and multivariate kurtosis were both reported with*p*<0.01, meeting the proposed threshold of *p* < 0.05 [76].

### 3.5 Data analysis method

For statistical analysis, the partial least squares structural equation modelling (PLS-SEM) approach and the program SmartPLS (version 3.5) were employed. The analysis was divided into two sections. Based on Hair et al. [77], the first phase was performed to evaluate the study model's reliability and validity using Cronbach's alpha, Dijkstra-Hensele's rho, composite reliability, and average variance extracted (AVE). To examine discriminant validity, Fornell-Larcker criterion, heterotrait-monotrait ratio (HTMT), and cross-loadings were used. The second step entailed assessing association between independent and dependent variables, includes analysing the significance levels, path coefficients and determining the mediating effects.

## 4 Findings

### 4.1 Respondents’ profile

The detailed profile of the 571 participants is presented in Table 1. First, 56.6% of the respondents were females while 43.4% were males. Second, most participants were aged 25–30 years old (52.4%), followed by 18–24 years old (47.6%). Regarding monthly income, most of the respondents earned between 2501 and 5000 Yuan (25.6%), followed by below 2500 Yuan (19.1%), and 5001 to 7500 Yuan (18.7%). Next, most respondents had a bachelor's degree or equivalent (68.3%), while 27.0% had a master's degree and 4.7% had a doctorate. For living area, most of the respondents resided in East China (33.8%), followed by North China (21.5%) and Central China (19.3%), whereas the remaining respondents were distributed across several other locations in China.

**Table 1:** Demographic characteristics.

	N	%			N	%
*Gender*				*Education*		
Male	248	43.4		Bachelor degree or equivalent	390	68.3
Female	323	56.6		Master degree	154	27.0
Total	571	100.0		Doctoral degree	27	4.7
				Total	571	100.0
*Age Group*						
18–24 years	272	47.6		*Respondents Location*		
25–30 years	299	52.4		East China	193	33.8
Total	571	100.0		Southern China	56	9.8
				Central China	110	19.3
*Average Monthly Income (Yuan)*				North China	123	21.5
Below 2500 Yuan	109	19.1		Northwest China	13	2.3
2501 to 5000 Yuan	146	25.6		Southwest China	60	10.5
5001 to 7500 Yuan	107	18.7		Northeast China	7	1.2
7500 to 10000 Yuan	64	11.2		Others	9	1.6
10001 to 12000 Yuan	69	12.1		Total	571	100.0
More than 12000 Yuan	76	13.3				
Total	571	100.0				

### 4.2 Validity and reliability

The internal consistency of components in a research model is typically assessed using Dijkstra-Hensele's rho, Cronbach's alpha, and composite reliability, whereby a threshold value of higher than 0.7 indicates strong internal consistency and reliability [77]. The Cronbach's alpha (0.833–0.939), Dijkstra-Hensele's rho (0.840–0.939), and composite reliability (0.882–0.953) values, as reported in Table 2, were higher than 0.7 for all the components and established strong reliability and internal consistency. The AVE of all items for each construct must be above a threshold of 0.50 [78] to assure that the model and its components have excellent convergent validity. In this study, the AVE values ranged from 0.600 to 0.804 (Table 2), indicating substantial convergent validity. On the other hand, multicollinearity issue was assessed using VIF. The VIF values reported in Table 2 were less than 5.0 for all the components, as recommended by Hair et al. [77], indicating that there was no multicollinearity issue in this study's dataset.

**Table 2:** Validity and reliability.

Variables	No. Items	Mean	Standard Deviation	Cronbach's Alpha	Dijkstra-Hensele's *rho*	Composite Reliability	Average Variance Extracted	Variance Inflation Factors
HV	4	6.126	0.978	0.876	0.879	0.915	0.730	1.819
HC	5	5.762	0.918	0.833	0.840	0.882	0.600	1.819
HE	5	5.598	1.142	0.903	0.905	0.930	0.729	1.870
AC	5	5.733	0.971	0.849	0.850	0.892	0.625	3.262
AR	5	5.907	0.992	0.911	0.911	0.933	0.737	2.514
PN	5	5.214	1.342	0.935	0.936	0.951	0.795	2.065
TOF	5	5.282	1.234	0.937	0.937	0.952	0.798	2.065
IOF	6	4.872	1.297	0.931	0.932	0.946	0.745	1.000
COF	5	4.805	1.482	0.939	0.939	0.953	0.804	–

**Note:** HV: Health Values, HC: Health Consciousness, HE: Healthy Eating Beliefs, AC: Awareness of Consequences, AR: Ascription of Responsibility, PN: Personal Norms; TOF: Trust on Organic Food, IOF: Intention towards Organic Food Consumption, COF: Consumption of Organic Food.

To gain a thorough explanation of the discriminant validity, the Fornell-Larcker criterion, the HTMT ratio, and cross-loadings were determined in this study. The square root of the AVE value must be higher than the variances of any other latent variables when evaluating the Fornell-Larcker criterion [78]. As listed in Table 3 (in bold), the square root of the AVE value of each component was found to be higher than any correlations in the column and row that contained it. On the other hand, according to Henseler et al. [79], all the HTMT values should be smaller than 0.90 to have good discriminant validity. Table 3 shows that the values of HTMT for all the components have satisfied the criterion. External loads greater than 0.7 are desirable [75]. In this study, the cross-loadings (as presented in Table 4) ranged from 0.674 (HE1) to 0.919 (TOF3), and all the cross-loading values were greater than 0.7 except for HE1. Nevertheless, the cross-loading of HE1 was between 0.4 and 0.7 and was the largest value in comparison with the squared loadings of other items. The item was retained when the previous composite reliability and convergent validity values were combined, and the indicator reliability of the measurement model existed. In short, all three analyses supported the components' strong discriminant validity in this current study's model.

**Table 3:** Discriminant validity.

	HV	HC	HE	AC	AR	PN	TOF	IOF	COF
*Fornell-Larcker Criterion*									
HV	0.854								
HC	0.671	0.774							
HE	0.434	0.563	0.854						
AC	0.594	0.697	0.681	0.790					
AR	0.584	0.651	0.552	0.775	0.859				
PN	0.327	0.475	0.640	0.570	0.516	0.891			
TOF	0.285	0.434	0.646	0.513	0.449	0.718	0.894		
IOF	0.179	0.322	0.513	0.361	0.277	0.665	0.736	0.863	
COF	0.179	0.338	0.528	0.355	0.292	0.704	0.731	0.817	0.896
*Heterotrait-Monotrait Ratio (HTMT)*									
HV									
HC	0.777								
HE	0.488	0.644							
AC	0.690	0.822	0.768						
AR	0.652	0.741	0.608	0.882					
PN	0.359	0.543	0.696	0.629	0.560				
TOF	0.314	0.497	0.702	0.565	0.486	0.766			
IOF	0.198	0.373	0.559	0.395	0.301	0.711	0.788		
COF	0.198	0.388	0.573	0.386	0.316	0.751	0.780	0.872	

**Note:** HV: Health Values, HC: Health Consciousness, HE: Healthy Eating Beliefs, AC: Awareness of Consequences, AR: Ascription of Responsibility, PN: Personal Norms; TOF: Trust on Organic Food, IOF: Intention towards Organic Food Consumption, COF: Consumption of Organic Food.

**Table 4:** Loading and cross-loading.

Code	HV	HC	HE	AC	AR	PN	TOF	IOF	COF
HV1	*0.802*	0.524	0.343	0.463	0.438	0.207	0.217	0.135	0.133
HV2	*0.870*	0.582	0.388	0.511	0.505	0.332	0.284	0.198	0.216
HV3	*0.882*	0.622	0.386	0.543	0.548	0.303	0.261	0.162	0.139
HV4	*0.860*	0.560	0.363	0.508	0.501	0.269	0.205	0.114	0.121
HC1	0.526	*0.745*	0.441	0.489	0.447	0.359	0.327	0.241	0.280
HC2	0.406	*0.708*	0.356	0.437	0.396	0.410	0.389	0.321	0.323
HC3	0.495	*0.807*	0.426	0.566	0.517	0.294	0.308	0.180	0.177
HC4	0.627	*0.817*	0.493	0.624	0.586	0.358	0.329	0.207	0.210
HC5	0.517	*0.790*	0.446	0.562	0.554	0.432	0.344	0.317	0.336
HE1	0.485	0.566	*0.674*	0.623	0.528	0.377	0.355	0.222	0.227
HE2	0.362	0.510	*0.904*	0.599	0.481	0.593	0.590	0.490	0.507
HE3	0.337	0.452	*0.893*	0.554	0.455	0.557	0.574	0.479	0.477
HE4	0.305	0.423	*0.887*	0.540	0.423	0.595	0.605	0.492	0.499
HE5	0.357	0.439	*0.887*	0.576	0.458	0.593	0.618	0.492	0.529
AC1	0.536	0.590	0.497	*0.799*	0.650	0.377	0.336	0.189	0.176
AC2	0.537	0.586	0.489	*0.831*	0.661	0.368	0.355	0.189	0.174
AC3	0.490	0.583	0.503	*0.840*	0.655	0.389	0.329	0.208	0.199
AC4	0.399	0.474	0.477	*0.736*	0.560	0.432	0.387	0.287	0.295
AC5	0.388	0.515	0.687	*0.739*	0.539	0.644	0.580	0.511	0.513
AR1	0.532	0.576	0.487	0.691	*0.857*	0.431	0.382	0.222	0.239
AR2	0.502	0.587	0.515	0.683	*0.865*	0.478	0.428	0.293	0.307
AR3	0.474	0.540	0.453	0.641	*0.863*	0.431	0.367	0.219	0.254
AR4	0.480	0.526	0.447	0.644	*0.846*	0.440	0.385	0.219	0.221
AR5	0.519	0.565	0.463	0.669	*0.861*	0.436	0.365	0.234	0.231
PN1	0.214	0.421	0.543	0.455	0.404	*0.904*	0.650	0.618	0.659
PN2	0.311	0.429	0.608	0.535	0.460	*0.901*	0.667	0.625	0.653
PN3	0.334	0.446	0.547	0.568	0.543	*0.851*	0.598	0.518	0.527
PN4	0.290	0.412	0.589	0.495	0.428	*0.915*	0.644	0.606	0.664
PN5	0.309	0.413	0.564	0.491	0.472	*0.885*	0.640	0.594	0.628
TOF1	0.227	0.406	0.611	0.435	0.395	0.688	*0.861*	0.668	0.666
TOF2	0.309	0.430	0.607	0.497	0.457	0.628	*0.891*	0.636	0.635
TOF3	0.231	0.375	0.584	0.453	0.401	0.654	*0.919*	0.675	0.667
TOF4	0.271	0.379	0.553	0.465	0.390	0.622	*0.907*	0.651	0.652
TOF5	0.237	0.351	0.531	0.443	0.366	0.613	*0.889*	0.657	0.645
IOF1	0.147	0.292	0.448	0.325	0.239	0.543	0.612	*0.868*	0.695
IOF2	0.154	0.265	0.443	0.304	0.232	0.596	0.640	*0.860*	0.722
IOF3	0.170	0.301	0.452	0.314	0.274	0.591	0.647	*0.858*	0.674
IOF4	0.196	0.319	0.437	0.364	0.284	0.522	0.638	*0.838*	0.635
IOF5	0.097	0.219	0.395	0.261	0.185	0.575	0.607	*0.877*	0.746
IOF6	0.168	0.275	0.481	0.308	0.227	0.613	0.668	*0.876*	0.753
OFC1	0.151	0.334	0.492	0.331	0.287	0.621	0.669	0.740	*0.904*
OFC2	0.225	0.350	0.504	0.382	0.341	0.605	0.696	0.712	*0.866*
OFC3	0.118	0.266	0.438	0.292	0.211	0.592	0.608	0.760	*0.903*
OFC4	0.116	0.257	0.458	0.272	0.208	0.654	0.656	0.729	*0.915*
OFC5	0.199	0.309	0.477	0.317	0.267	0.684	0.651	0.722	*0.893*

**Note:** HV: Health Values, HC: Health Consciousness, HE: Healthy Eating Beliefs, AC: Awareness of Consequences, AR: Ascription of Responsibility, PN: Personal Norms; TOF: Trust on Organic Food, IOF: Intention towards Organic Food Consumption, COF: Consumption of Organic Food.

### 4.3 Path analysis

Table 5 presents the results of the path analysis and hypothesis testing obtained through the bootstrapping technique. The path coefficients and *p*-values of HV (β = 0.103, *p*-value = 0.025) and HC (β = 0.494, *p*-value = 0.000) were positive and statistically significant. Both HV and HC have a significant and favourable impact on HE. Thus, hypotheses H1 and H2 are supported. Results for the relationship between HE and AC were a β-value of 0.681 and a *p*-value of 0.000. The outcome indicates that HE has a substantial positive influence on AC, and this supports hypothesis H3a. Next, the relationship between AC and AR was also confirmed by a positive β-value (0.775) and a significant *p*-value (0.000). This demonstrates that AC has a significant and beneficial effect on AR, hence, confirming hypothesis H4a. The fourth section of Table 5 shows the correlation between PN and HE (β = 0.463, *p*-value = 0.000), AC (β = 0.132, *p*-value = 0.018), and AR (β = 0.159, *p*-value = 0.005), all of which exhibit positive path coefficients and statistically significant *p*-values. These findings corroborate hypotheses H3b, H4b, and H5 by confirming that HE, AC, and AR have a favourable and significant effect on PN. In the fifth section of Table 5, the relationships between IOF and PN and IOF and TOF are established with positive path coefficients (PN = 0.282, TOF = 0.534) and significant *p*-values (PN = 0.000, TOF = 0.000). These results support hypotheses H6 and H7. Finally, a β-value of 0.817 and a *p*-value of 0.000 were noted for the relationship between IOF and COF. Hence, the analysis has established that IOF has a strong positive effect on COF, and hypothesis H8 is validated.

**Table 5:** Hypotheses testing.

Hypo		Beta	CI Min	CI Max	*t Value*	*p value*	*R*^2^	*f*^*2*^	*Q*^*2*^	Decision
H_1_	HV → HE	0.103	0.015	0.189	1.962	0.025		0.009		Accept
H_2_	HC → HE	0.494	0.409	0.580	9.800	0.000	0.322	0.198	0.222	Accept
H_3a_	HE → AC	0.681	0.633	0.731	21.884	0.000	0.464	0.866	0.278	Accept
H_4a_	AC → AR	0.775	0.724	0.815	28.289	0.000	0.601	1.507	0.440	Accept
H_3b_	HE → PN	0.463	0.379	0.565	8.251	0.000		0.210		Accept
H_4b_	AC → PN	0.132	0.024	0.236	2.114	0.018	0.453	0.018	0.357	Accept
H_5_	AR → PN	0.159	0.054	0.262	2.561	0.005		0.010		Accept
H_6_	PN → IOF	0.282	0.196	0.364	5.759	0.000		0.092		Accept
H_7_	TOF → IOF	0.534	0.445	0.620	10.368	0.000	0.581	0.328	0.429	Accept
H_8_	IOF → COF	0.817	0.781	0.847	41.029	0.000	0.668	2.013	0.533	Accept

**Note:** HV: Health Values, HC: Health Consciousness, HE: Healthy Eating Beliefs, AC: Awareness of Consequences, AR: Ascription of Responsibility, PN: Personal Norms; TOF: Trust on Organic Food, IOF: Intention towards Organic Food Consumption, COF: Consumption of Organic Food.

The Q^2^ test is employed for a more in-depth investigation of the predictive relevance of endogenous components [75]. A Q^2^ greater than zero indicates significant predictive relevance [78]. In the current study, the Q^2^ values (HE = 0.222, AC = 0.278, AR = 0.440, PN = 0.357, IOF = 0.429, and COF = 0.533) were above the threshold value, signifying strong predictive relevance. Furthermore, the coefficient of determination (*R*^*2*^) denotes the degree of explained variances, which is the proportion of the variation in the dependent variable explained by a linear model. Significant, moderate, or weak endogenous latent variables have r^2^ values of 0.75, 0.50, or 0.25, respectively [77]. Based on the *R*^*2*^ values reported in Table 5 (HE = 0.322, AC = 0.464, AR = 0.601, PN = 0.453, IOF = 0.581, and COF = 0.668), all the predictors demonstrated moderate explanatory power in this current study.

### 4.4 Indirect effects

Besides the direct association between constructs, Hair et al. [77] have advised that the indirect effects of all the correlations should be investigated. Table 6 reports the analysis of the mediation effects of HE, PN, and IOF. According to the findings, the relevant components designed in the study model fully mediated all the associations, and every indirect effect was significant with positive path coefficients.

**Table 6:** Indirect effects.

	Beta	CI Min	CI Max	*t Value*	*p value*
Health Value - > Ascription of Responsibility	0.054	0.007	0.104	1.896	0.029
Health Value - > Awareness of Consequences	0.070	0.010	0.131	1.935	0.027
Health Value - > Personal Norms	0.065	0.010	0.119	1.965	0.025
Health Value - > Intention towards Organic Food Consumption	0.018	0.002	0.035	1.859	0.032
Health Value - > Consumption of Organic Food	0.015	0.002	0.029	1.869	0.031
Health Consciousness - > Ascription of Responsibility	0.261	0.206	0.325	7.408	0.000
Health Consciousness - > Awareness of Consequences	0.336	0.267	0.405	8.145	0.000
Health Consciousness - > Personal Norms	0.314	0.254	0.384	7.838	0.000
Health Consciousness - > Intention towards Organic Food Consumption	0.089	0.057	0.126	4.398	0.000
Health Consciousness - > Consumption of Organic Food	0.072	0.047	0.104	4.341	0.000
Healthy Eating Belief - > Ascription of Responsibility	0.528	0.467	0.586	14.835	0.000
Healthy Eating Belief - > Personal Norms	0.174	0.117	0.233	4.890	0.000
Healthy Eating Belief - > Intention towards Organic Food Consumption	0.180	0.125	0.237	5.382	0.000
Healthy Eating Belief - > Consumption of Organic Food	0.147	0.102	0.195	5.292	0.000
Awareness of Consequences - > Ascription of Responsibility	0.123	0.041	0.209	2.513	0.006
Awareness of Consequences - > Intention towards Organic Food Consumption	0.072	0.042	0.103	3.917	0.000
Awareness of Consequences - > Consumption of Organic Food	0.059	0.035	0.085	3.952	0.000
Ascription of Responsibility - > Intention towards Organic Food Consumption	0.045	0.016	0.080	2.342	0.010
Ascription of Responsibility - > Consumption of Organic Food	0.037	0.013	0.065	2.339	0.010
Personal Norms - > Consumption of Organic Food	0.231	0.157	0.297	5.697	0.000
Trust on Organic Food - > Consumption of Organic Food	0.436	0.357	0.511	9.558	0.000

## 5 Discussion

The current study used the VBN theory to investigate the associations among healthy eating beliefs, health values, and health consciousness, besides the correlations between personal norms and health consciousness, awareness of consequences, and ascription of responsibility. This study also examined the relationships between intention to consume organic foods and personal norms and trust in organic food, as well as the association between consumption of organic foods and intention to consume organic foods. Using the SEM analysis, all the relationships were identified to be positive and significant, and all the mediators were reported to have substantial mediating impacts on the corresponding associations in the proposed model. The obtained results are scrutinised based on existing literature as follows.

First, HV substantially affected HE. This result is consistent with studies by Liu et al. [5] and Nafees et al. [4], who have empirically demonstrated that HV plays a major role in organic buying behaviour. It indicates that nowadays people are prioritising a healthier lifestyle and becoming more health-literate. The finding also denotes that those consumers evaluate organic food for its high health benefits towards gaining good health. Similarly, HC also positively and substantially influenced HE. This outcome is in line with findings in numerous earlier studies [12,19]. The current study's finding shows that consumers who firmly adhere to self-care and possess strong health awareness are more likely to relate organic food to healthy eating beliefs.

Next, HE was positively associated with AC and PN. These findings align with the study of Van Riper & Kyle [22] on natural resources and the study of Fielding-Singh [39] on healthy eating behaviour. It signifies that healthy foods are preferable to unhealthy foods among organic food customers, whereby such a belief might motivate people to develop more awareness about the benefits of organic foods. This study also found that both AC and AR had a considerable influence on PN. The findings correspond to those of Choi et al. [65] in the context of green hotels and Fatemi and Rezaei-Moghaddam [24] in the context of organic farming. The analyses indicate that customers who are concerned about the adverse issue of unhealthy and conventional food practices are more likely to feel morally and intuitively obligated to switch to organic foods.

Another key finding of the present study was that PN and TOF had positive and significant impacts on consumers' IOF. This finding concurs with those in previous studies in the context of organic food consumption and its purchasing behaviour [10]. The results show that those who feel morally obligated for eating healthy food and feel liable for the environmental repercussions of non-organic food are more likely to grow a strong intention to consume organic foods. At the same time, trust in organic food, such as its esteemed health benefits and nutritious values, ethical and environmentally friendly techniques of production, and naturalness, is an equally vital aspect in enhancing the intention to consume organic foods. Besides, the intention to consume organic foods influenced actual consumption. This is consistent with previous studies’ findings in the context of organic farming and organic food purchasing behaviour [24,54,80]. Finally, according to the research model, all the mediators (i.e., healthy eating beliefs, personal norms, and intention to consume organic foods) positively and significantly mediated all the relevant associations. A reasonable interpretation of such results is that consumers with a strong intention to consume organic foods are more likely to be the obvious consumers of organic foods, and their pro-environmental and healthier life related values, beliefs, and norms play the most influential role in making that decision.

## 6 Implications

### 6.1 Theoretical implication

This study gives an insight into an unexplored theoretical basis of organic food consumers' decision making and presents a theoretical framework that contributes to the current literature on organic food consumption behaviour. The study framework supports the VBN theory and has expanded it with three comparatively unexplored factors (i.e., health values, healthy eating beliefs, and trust in organic food) to better understand the effects of healthier living related beliefs and norms and how these are related to the consumption behaviour of organic foods. By empirically evaluating the proposed expanded VBN model in the context of organic food consumption in a developing country and among young adults, the current study has contributed to the existing literature by confirming the effective applicability of the VBN theory. The empirical investigation demonstrated that all the components of the VBN model had a robust effect on consumers’ organic food consumption.

### 6.2 Practical and managerial implications

According to the findings of this study, health values and health consciousness are decisive indicators of the growing healthy eating beliefs towards organic foods. Hence, it is recommended that marketers should properly convey the expected health benefits of organic foods through advertising initiatives in both electronic and print media. Dietitians may present customers with additional product information via media and social networks to highlight the beneficial differences between organic food and conventional food. Besides, organic merchants should focus on certain groups of consumers to increase customers' values regarding green and organic foods while retaining their happiness and enjoyment. Simultaneously, public policymakers can deliver more authoritative information and organic standards to the general public via social platforms and all other media, therefore, supporting organic food consumption and enhancing consumers’ values in sustainable development.

Healthy eating beliefs favourably affect personal norms and awareness of consequences. As such, manufacturers of organic food should develop labels and packaging that highlight these products' special features. Consequently, product labels may assist consumers in developing an optimistic perception and thinking rationally while making a purchase. Furthermore, being consistent about the claims of special features might increase customers' trust towards the related organic brands and induce consumers' strong affection for them. Therefore, maintaining strict quality standards is a crucial factor for manufacturers to gain the trust of consumers, which they must constantly retain. Concurrently, public policymakers should inaugurate education programmes and publicity to circulate the standards of organic and green farming and breeding that are favourable to biological sustainability and public health, as well as enhance consumers' morals in supporting the growth of the organic food industry. Marketing experts and practitioners must constantly highlight and reaffirm that organic foods may contribute to the improvement of the consumers' family members, especially children's health, hence, increasing their favourable beliefs of organic foods.

Given that trust in organic food is a leading factor for the intention to consume organic foods, knowledge alone is not enough to convince the consumers to consume organic foods if they are doubtful regarding the purported advantages. Therefore, advertisers and practitioners must ensure that their assertions of naturalness are substantiated by providing precise information about the ingredients and formulations of their goods. If their products are certified by a trustworthy certifying agency and government regulatory bodies, they can generally be considered trustworthy. To ensure the integrity of organic foods, several marketing tactics are thus recommended to be implemented. The tactics are offering trial bundle packs, offering money back if products are deemed as being of questionable quality, and selling items in smaller volumes so that consumers feel that waste would be limited, even though the products do not provide the expected benefits. Henceforth, government and policymakers should enforce clear rules and certifications and establish a continuous monitoring system to ensure that manufacturers are meeting the standards at all times.

Finally, producers and marketers of organic foods should collaborate with non-profit activist groups and a variety of social media communities to raise consumer awareness of ecological adversity by highlighting the adverse impacts conventional food habits have on the climate.

## 7 Conclusion

The conventional food production system, which is built on heavy agrochemicals including chemical fertilizers and pesticides, has recently received criticism [24]. On the other hand, organic farming is regarded as one of the most effective alternative farming technologies for producing organic food free of chemicals. The current study intended to fill significant gaps in the literature by investigating the socio-psychological behavioural factors of young Chinese organic food consumers. A cross-sectional study collecting 571 consumers' data was performed to evaluate the proposed framework, which underpinned the theoretical framework of VBN. The model demonstrated significant explanatory power, featuring justifications for purchasing organic foods and their environmental advantages resulting in health values, healthy eating beliefs, personal norms, and trust as the strongest predictors. The statistical analysis revealed support for all the hypotheses and all the indirect effects among the projected associations. This study's findings revealed intriguing and impelling information regarding the decision-making factors of consumers. Manufacturers and marketers of organic food must emphasise the impact of different consumers' values, beliefs, and norms on practical consumption behaviour to reflect different consumer choices. In emphasising organic food production standards, besides providing a description of the nutrition content and improving organic labelling, manufacturers should also focus on getting certification from trustworthy private and government agencies to gain consumers' trust. This study's findings will also be useful for the government, policymakers, and organic producers, dealers, and retailers, to better understand the impact of customers' insights and conventions on their purchasing intentions of other organic and eco-friendly products.

Some limitations of the current study point to future research directions. First, the study data were gathered from organic food consumers in China's universities, which may restrict the study's generalisability to other circumstances. The results may differ from the findings for other age groups or for populations living in non-Tier 1 urban areas. Thus, future investigations with a larger number of respondents from diverse demographics and locations may assist to enhance the understanding of organic food consumption behaviour and increase the generalisability of the study framework. Second, the study only addressed a few factors related to values, beliefs, and norms, and may have overlooked additional crucial elements (such as price value and subjective norms). University students are often more idealistic and relatively more exposed to the concept of organic food and green consumerism, and their health values, health awareness and other factors may be more positively inclined towards green consumption. However, for the rest of the population, factors such as income, price value, ease of purchase and subjective norms may also be influencing organic food consumption. As such, future research may consider other factors, and thereby, enrich the results.

## Authors contribution

Farzana Naznen, Long Siyu and Zafir Khan Mohamed Makhbul: Conceived and designed the experiments; Performed the experiments; Wrote the paper. Abdullah Al Mamun and Qing Yang: Performed the experiments; Analyzed and interpreted the data; Contributed reagents, materials, analysis tools or data; Wrote the paper. All authors approved the final version of the manuscript and give their consent for submission and publication.

## Availability of data and materials

The original contributions presented in the study are included in the article/Supplementary Material (S2. Dataset), further inquiries can be directed to the corresponding author/s.

## Funding

This research received no specific grant from any funding agency in the public, commercial, or not-for-profit sectors.

## Declaration of competing interest

The authors declare that they have no known competing financial interests or personal relationships that could have appeared to influence the work reported in this paper.

## References

[1] Murphy B., Martini M., Fedi A., Loera B.L., Elliott C.T., Dean M. (2022). Consumer Trust in Organic Food and organic certifications in four European countries. Food Control.

[2] World Health Organization (2021). Noncommunicable diseases. https://www.who.int/news-room/fact-sheets/detail/noncommunicable-diseases.

[3] Muhie S.H. (2022). Novel approaches and practices to sustainable agriculture. Journal of Agriculture and Food Research.

[4] Nafees L., Hyatt E.M., Garber L.L., Das N., Boya Ü.Ö. (2022). Motivations to buy organic food in emerging markets: an exploratory study of urban Indian millennials. Food Qual. Prefer..

[5] Liu C., Zheng Y., Cao D. (2021). Similarity effect and purchase behavior of organic food under the mediating role of perceived values in the context of COVID-19. Front. Psychol..

[6] Molinillo S., Vidal-Branco M., Japutra A. (2020). Understanding the drivers of organic foods purchasing of millennials: evidence from Brazil and Spain. J. Retailing Consum. Serv..

[7] Tandon A., Dhir A., Kaur P., Kushwah S., Salo J. (2020). Why do people buy organic food? the moderating role of environmental concerns and Trust. J. Retailing Consum. Serv..

[8] Willer H., Schlatter B., Trávníček J., Kemper L., Lernoud J. (2020). Research Institute of Organic Agriculture (FiBL) and IFOAM – Organics International.

[9] Global Organic Trade Guide (2023). China: sustainability is one of the factors for purchase decisions. Global Organic Trade Guide.

[10] Hansmann R., Baur I., Binder C.R. (2020). Increasing organic food consumption: an integrating model of drivers and barriers. J. Clean. Prod..

[11] Nicolopoulou-Stamati P., Maipas S., Kotampas C., Stamatis P., Hens L. (2016). Chemical pesticides and human health: the urgent need for a new concept in agriculture. Front. Public Health.

[12] Iqbal J., Yu D., Zubair M., Rasheed M.I., Muhammad H., Khizar U., Imran M. (2021).

[13] Popa M.E., Mitelut A.C., Popa E.E., Stan A., Popa V.I. (2019). Organic foods contribution to nutritional quality and value. Trends Food Sci. Technol..

[14] Van Bussel L.M., Kuijsten A., Mars M., vantVeer P. (2022). Consumers' perceptions on food-related sustainability: a systematic review. J. Clean. Prod..

[15] Sreen N., Dhir A., Talwar S., Ming T., Alharbi F. (2021). Behavioral reasoning perspectives to brand love toward natural products : moderating role of environmental concern and household size. J. Retailing Consum. Serv..

[16] Pocol C.B., Marinescu V., Dabija D.-C., Amuza A. (2021). Clustering generation Z university students based on daily fruit and vegetable consumption: empirical research in an emerging market. Br. Food J..

[17] Kushwah S., Dhir A., Sagar M. (2019). Ethical consumption intentions and choice behavior towards organic food. moderation role of buying and environmental concerns. J. Clean. Prod..

[18] Shamsi H.R., Najafabadi M.O., Hosseini S.J. (2020). Designing a three-phase pattern of organic product consumption behaviour. Food Qual. Prefer..

[19] Nguyen H.V., Nguyen N., Nguyen B.K., Lobo A. (2019). Organic food purchases in an emerging market : the influence of consumers' personal factors and green marketing practices of food stores. International Journal of Environmental Research and Public Health, MDPI.

[20] Channa N.A., Tariq B., Samo A.H., Ghumro N.H., Qureshi N.A. (2021). Predicting consumers' intentions to purchase eco-friendly athletic wear in a moderated model of individual green values and gender. Int. J. Sports Mark. Spons..

[21] Tandon A., Jabeen F., Talwar S., Sakashita M., Dhir A. (2021). Facilitators and inhibitors of organic food buying behavior. Food Qual. Prefer..

[22] Van Riper C.J., Kyle G.T. (2014). Understanding the internal processes of behavioral engagement in a national park: a latent variable path analysis of the value-belief-norm theory. J. Environ. Psychol..

[23] Stern P.C., Dietz T., Abel T., Guagnano G.A., Kalof L. (1999). A value-belief-norm theory of support for social movements: the case of environmentalism. Hum. Ecol. Rev..

[24] Fatemi M., Rezaei-Moghaddam K. (2020). Sociological factors influencing the performance of organic activities in Iran. Life Sciences, Society and Policy.

[25] Ünal A.B., Steg L., Granskaya J. (2019). To support or not to support, that is the question”. testing the VBN theory in predicting support for car use reduction policies in Russia. Transport. Res. Pol. Pract..

[26] Han H. (2020). Theory of green purchase behavior (TGPB): a new theory for sustainable consumption of Green Hotel and Green Restaurant Products. Bus. Strat. Environ..

[27] Jaini A., Quoquab F., Mohammad J., Hussin N. (2020). Antecedents of green purchase behavior of cosmetics products. International Journal of Ethics and Systems.

[28] Dang V.T., Wang J., Nguyen H.V., Nguyen Q.H., Nguyen N. (2021). A moderated mediation study of consumer extrinsic motivation and CSR beliefs towards organic drinking products in an emerging economy. Br. Food J..

[29] Schwartz S.H. (1992). Universals in the content and structure of values: theoretical advances and empirical tests in 20 countries. Adv. Exp. Soc. Psychol..

[30] Lau R.R., Hartman K.A., Ware J.E. (1986). Health as a value: methodological and theoretical considerations. Health Psychol..

[31] Chakraborty D., Siddiqui A., Siddiqui M., Mohmmad H Alatawi F. (2022). Exploring consumer purchase intentions and behavior of buying ayurveda products using SOBC framework. J. Retailing Consum. Serv..

[32] Mohammed A.A. (2020). What motivates consumers to purchase organic food in an emerging market? an empirical study from Saudi Arabia. Br. Food J..

[33] Yang Q., Al Mamun A., Hayat N., Salleh Md, Salameh A.A., Makhbul Z.K. (2022). Predicting the mass adoption of eDoctor apps during COVID-19 in China using hybrid sem-neural network analysis. Front. Public Health.

[34] Xinyan Z., Mamun A.A., Ali M.H., Siyu L., Yang Q., Hayat N. (2022). Modeling the adoption of medical wearable devices among the senior adults: using hybrid sem-neural network approach. Front. Public Health.

[35] Leanh T., NguyenTo T. (2020). Consumer purchasing behaviour of organic food in an emerging market. Int. J. Consum. Stud..

[36] Rana J., Paul J. (2020). Health motive and the purchase of Organic Food: a meta‐analytic review. Int. J. Consum. Stud..

[37] Rana J., Paul J. (2017). Consumer behavior and purchase intention for organic food: a review and research agenda. J. Retailing Consum. Serv..

[38] Heijden A. Van Der, Jager G., Mulder B.C. (2021). Healthy eating beliefs and the meaning of food in populations with a low socioeconomic position: a scoping review. Appetite.

[39] Fielding-Singh P. (2019). You're worth what you eat: adolescent beliefs about healthy eating, morality and socioeconomic status. Soc. Sci. Med..

[40] De Groot J.I., Steg L. (2009). Morality and prosocial behavior: the role of awareness, responsibility, and norms in the norm activation model. J. Soc. Psychol..

[41] Nguyen T.P., Doan X.H., Nguyen T.T., Nguyen T.M. (2021). Factors affecting Vietnamese farmers' intention toward organic agricultural production. Int. J. Soc. Econ..

[42] Roos D., Hahn R. (2019). Understanding collaborative consumption: an extension of the theory of planned behavior with value-based personal norms. J. Bus. Ethics.

[43] Sultan P., Tarafder T., Pearson D., Henryks J. (2020). Intention-behaviour gap and perceived behavioural control-behaviour gap in theory of planned behaviour: moderating roles of communication, satisfaction and trust in Organic Food Consumption. Food Qual. Prefer..

[44] Al Mamun A., Hayat N., Masud M.M., Yang Q., Salameh A.A., Salleh M.F. (2022). Energy conservation behaviour among the Malaysian youth: a study under the premises of value-belief-norm model. Front. Energy Res..

[45] Duong C.D., Doan X.H., Vu D.M., Ha N.T., Dam K.V. (2022). The role of perceived environmental responsibility and environmental concern on shaping green purchase intention. Vision: The Journal of Business Perspective.

[46] Doanh N.K., Quynh N.N., Pham T.T. (2022). Going organic or staying traditionalistic? The role of agriculture information system. Int. J. Soc. Econ..

[47] Hoeksma D.L., Gerritzen M.A., Lokhorst A.M., Poortvliet P.M. (2018). An extended theory of planned behavior to predict consumers' willingness to buy mobile slaughter unit meat. Meat Sci..

[48] Han H., Hwang J., Lee M.J. (2016). The value–belief–emotion–norm model: investigating customers' eco-friendly behavior. J. Trav. Tourism Market..

[49] D'Arco M., Marino V. (2022). Environmental citizenship behavior and sustainability apps: an empirical investigation. Transforming Gov. People, Process Policy.

[50] Udo G.G., Bagchi K.K. (2019). The role of personal norm in predicting intention for digital piracy. Issues in Information Systems.

[51] D'Arco M., Marino V., Resciniti R. (2023). Exploring the pro-environmental behavioral intention of generation Z in the tourism context: the role of injunctive social norms and personal norms. J. Sustain. Tourism.

[52] Prakash G., Pathak P. (2017). Intention to buy eco-friendly packaged products among young consumers of India: a study on developing nation. J. Clean. Prod..

[53] Lazaroiu G., Andronie M., Uţă C., Hurloiu I. (2019). Trust management in organic agriculture: sustainable consumption behavior, environmentally conscious purchase intention, and Healthy Food Choices. Front. Public Health.

[54] Testa F., Sarti S., Frey M. (2018). Are green consumers really green? exploring the factors behind the actual consumption of organic food products. Bus. Strat. Environ..

[55] Roh T., Seok J., Kim Y. (2022). Unveiling ways to reach organic purchase: green perceived value, perceived knowledge, attitude, subjective norm, and Trust. J. Retailing Consum. Serv..

[56] Shahabi A.S., Gorton M. (2020). The effects of perceived regulatory efficacy, ethnocentrism and food safety concern on the demand for Organic Food. Int. J. Consum. Stud..

[57] Zhang Y., Jing L., Bai Q., Shao W., Feng Y., Yin S., Zhang M. (2018). Application of an integrated framework to examine Chinese consumers' purchase intention toward Genetically Modified Food. Food Qual. Prefer..

[58] Loebnitz N., Aschemann-Witzel J. (2016). Communicating Organic Food Quality in China: consumer perceptions of organic products and the effect of environmental value priming. Food Qual. Prefer..

[59] Laureti T., Benedetti I. (2018). Exploring pro-environmental food purchasing behaviour: an empirical analysis of Italian consumers. J. Clean. Prod..

[60] Yu W., Han X., Ding L., He M. (2021). Organic food corporate image and customer co-developing behavior: the mediating role of consumer trust and purchase intention. J. Retailing Consum. Serv..

[61] Stern P.C. (2000). New environmental theories: toward a coherent theory of environmentally significant behavior. J. Soc. Issues.

[62] Al Mamun A., Naznen F., Jingzu G., Yang Q. (2023). Predicting the intention and adoption of hydroponic farming among Chinese urbanites. Heliyon.

[63] Arab Rahmatipour M., Ebadollahi-Natanzi A., Arab-Rahmatipour G. (2020). Letter to the editor: prevention of depression and psychological stress by studying book in quarantine conditions of covid-19. SciMedicine Journal.

[64] Faul F., Erdfelder E., Lang A.-G., Buchner A. (2007). G*Power 3: a flexible statistical power analysis program for the social, Behavioral, and Biomedical Sciences. Behav. Res. Methods.

[65] Choi H., Jang J., Kandampully J. (2015). Application of the extended VBN theory to understand consumers' decisions about Green Hotels. Int. J. Hospit. Manag..

[66] Chen Y.-S. (2009). The drivers of green brand equity: green brand image, green satisfaction, and green trust. J. Bus. Ethics.

[67] Chen K., Deng T. (2016). Research on the Green purchase intentions from the perspective of product knowledge. Sustainability.

[68] Maichum K., Parichatnon S., Peng K.-C. (2016). Application of the extended theory of planned behavior model to investigate purchase intention of green products among Thai consumers. Sustainability.

[69] Walton T., Austin D.M. (2011). Pro-environmental behavior in an urban social structural context. Socio. Spectr..

[70] Sánchez M., López-Mosquera N., Lera-López F. (2015). Improving pro-environmental behaviours in Spain. the role of attitudes and socio-demographic and political factors. J. Environ. Pol. Plann..

[71] Chang S.J., van Witteloostuijn A., Eden L. (2010). From the Editors: common method variance in international business research. J. Int. Bus. Stud..

[72] Podsakoff P.M., MacKenzie S.B., Podsakoff N.P. (2012). Sources of method bias in social science research and recommendations on how to control it. Annu. Rev. Psychol..

[73] Sarstedt M., Ringle C.M., Hair J.F. (2017). Partial least squares structural equation modeling. Handbook of Market Research.

[74] Kock N., Lynn G. (2012). Lateral collinearity and misleading results in variance-based SEM: an illustration and recommendations. J. Assoc. Inf. Syst. Online.

[75] Hair J.F., Ringle C.M., Sarstedt M. (2011). PLS-SEM: indeed, a silver bullet. J. Market. Theor. Pract..

[76] Cain M.K., Zhang Z., Yuan K.-H. (2017). Univariate and multivariate skewness and kurtosis for measuring nonnormality: prevalence, influence and estimation. Behav. Res. Methods.

[77] Hair J.F., Risher J.J., Sarstedt M., Ringle C.M. (2019). When to use and how to report the results of PLS-SEM. Eur. Bus. Rev..

[78] Hair J.F., Hult G.T.M., Ringle C.M., Sarstedt M. (2017).

[79] Henseler J., Sarstedt M. (2012). Goodness-of-fit indices for partial least squares path modeling. Comput. Stat..

[80] Kashif U., Hong C., Naseem S. (2021). Assessment of millennial organic food consumption and moderating role of food neophobia in Pakistan Assessment of millennial organic food consumption and moderating role of food neophobia in Pakistan. Current Psychology, Springer.

