# Supplementary material for: Modelling the significance of health values, beliefs and norms on the intention to consume and the consumption of organic foods
Source: Heliyon. 2023 Jun 20;9(6):e17487. doi: 10.1016/j.heliyon.2023.e17487 (PMC10320173; doi:10.1016/j.heliyon.2023.e17487)
Supplement: Multimedia component 1 [file mmc1.docx]

**Appendix 1.** Survey Instrument

| Code | | Items | | |
| --- | --- | --- | --- | --- |
| HV1 | | If I don’t have my health, I don’t have anything | | |
| HV2 | | There is nothing I care more about than my health | | |
| HV3 | | Good health is most important for happy life | | |
| HV4 | | Nothing is more important than good health | | |
| HC1 | | I think my health depends on how well I take care of myself | | |
| HC2 | | I am actively engaged in the prevention of disease and illness | | |
| HC3 | | I think taking preventive measures help to stay healthy | | |
| HC4 | | Living a healthy life is important to me | | |
| HC5 | | I am constantly watchful about my health | | |
| HE1 | I believe eating plenty of vegetables, fruits, and whole grains good for my health | | | |
| HE2 | I believe eating organic food can reduce cancer risk | | | |
| HE3 | I believe eating organic food can improve gut health | | | |
| HE4 | I believe eating organic food can improve diabetes management | | | |
| HE5 | I believe eating organic food can prevent many health issues | | | |
| Awareness of Consequences | | | | |
| AC1 | Personal health management can improve the individual’s quality of life. | | | |
| AC2 | Adaptation of healthy eating practices can reduce the negative consequences towards personal health. | | | |
| AC3 | Health issues can be curtailed with the management of personal diet | | | |
| AC4 | Minor health concerns may lead to grave health issues | | | |
| AC5 | Overall, eating organic food can improve personal health. | | | |
| AR1 | I think taking responsibility for personal health is important. | | | |
| AR2 | I feel that taking responsibility for personal healthcare can help to promote a healthy society. | | | |
| AR3 | Taking personal responsibility for healthcare to promote personal wellbeing. | | | |
| AR4 | Everyone must take responsibility for personal health. | | | |
| AR5 | I feel responsible for personal healthcare. | | | |
| PN1 | I feel morally obliged to consume organic food | | | |
| PN2 | People like me should do everything they can to eat organic food | | | |
| PN3 | I feel obliged to bear the environment and nature in mind in my food consumption behaviours | | | |
| PN4 | I feel morally obliged to consume organic products, regardless of what others do | | | |
| PN5 | I feel personally obliged to promote organic food and organic food production methods | | | |
| TOF1 | | | Organic foods are generally reliable |  |
| TOF2 | | | Organic food’s environmental performance is generally dependable |  |
| TOF3 | | | Organic foods are generally trustworthy |  |
| TOF4 | | | Organic foods meets my expectations |  |
| TOF5 | | | Organic production method’s keep promises for environmental protection |  |
| IOF1 | I am willing to pay more for organic foods | | |  |
| IOF2 | I am willing to accept inconvenience to find organic food | | |  |
| IOF3 | I am willing to maximize the use of organic food and materials | | |  |
| IOF4 | I am willing to purchase organic food products manufactured or grown in an environmentally friendly ways | | |  |
| IOF5 | I am willing to pay premium price for organic food | | |  |
| IOF6 | I will encourage my friends and relatives to buy organic foods | | |  |
| OFC1 | I frequently consume organic food | | |  |
| OFC2 | I intentionally purchase organic food grown in an energy efficient environment | | |  |
| OFC3 | I frequently pay premium price for organic food | | |  |
| OFC4 | I set a positive example by consuming organic food for my community | | |  |
| OFC5 | I encourage my friends and relatives to consume organic food | | |  |
